# Phytochemical Discrimination, Biological Activity and Molecular Docking of Water-Soluble Inhibitors from *Saussurea costus* Herb against Main Protease of SARS-CoV-2

**DOI:** 10.3390/molecules27154908

**Published:** 2022-08-01

**Authors:** Hajo Idriss, Babeker Siddig, Pamela González Maldonado, H. M. Elkhair, A. I. Alakhras, Emad M. Abdallah, Pablo Hernán Sotelo Torres, Amin O. Elzupir

**Affiliations:** 1 Deanship of Scientific Research, Imam Mohammad Ibn Saud Islamic University (IMSIU), P.O. Box 90905, Riyadh 11623, Saudi Arabia; hiidriss@imamu.edu.sa (H.I.); hmdirar@imamu.edu.sa (H.M.E.); aakhrasi@imamu.edu.sa (A.I.A.); 2 Department of Physics, College of Science, Imam Mohammad Ibn Saud Islamic University (IMSIU), P.O. Box 90905, Riyadh 11623, Saudi Arabia; 3 Alawia Imam Institute for Pharmaceutical Research and Development, University of Medical Science and Technology, Khartoum 11115, Sudan; gcfid@savola.com; 4 Savola Edible Oils, Khartoum 11115, Sudan; 5 Biotechnology Department, Facultad de Ciencias Químicas, Universidad Nacional de Asunción, San Lorenzo 111421, Paraguay; pame.54.pg12@gmail.com (P.G.M.); phsotelo@qui.una.py (P.H.S.T.); 6 Department of Chemistry, College of Science, Imam Mohammad Ibn Saud Islamic University (IMSIU), Riyadh 11623, Saudi Arabia; 7 Department of Science Laboratories, College of Science and Arts, Qassim University, Ar Rass 51921, Saudi Arabia; emad100sdl@yahoo.com

**Keywords:** coronavirus SARS-CoV-2, COVID-19, main protease, *Saussurea costus*, molecular docking, GC-MS profiling

## Abstract

Siddha medicine is one of the oldest medical systems in the world and is believed to have originated more than 10,000 years ago and is prevalent across ancient Tamil land. It is undeniable that inhibitor preferences rise with increasing solubility in water due to the considerations pertaining to the bioavailability and the ease of which unabsorbed residues can be disposed of. In this study, we showed the phytochemical discrimination of *Saussurea costus* extracted with water at room temperature as a green extraction procedure. A total of 48 compounds were identified using gas chromatography-mass spectrometry (GC-MS). The fatty acids had a high phytochemical abundance at 73.8%, followed by tannins at 8.2%, carbohydrates at 6.9%, terpenoids at 4.3%, carboxylic acids at 2.5%, hydrocarbons at 2.4%, phenolic compounds at 0.2%, and sterols at 1.5%. Of these compounds, 22 were docked on the active side and on the catalytic dyad of His41 and Cys145 of the main protease of SARS-CoV-2 (M^pro^). Eight active inhibitors were carbohydrates, five were fatty acids, three were terpenoids, two were carboxylic acids, one was a tannin, one was a phenolic compound, and one was a sterol. The best inhibitors were 4,8,13-Cyclotetradecatriene-1,3-diol, 1,5,9-trimethyl-12-(1-methylethyl), Andrographolide, and delta.4-Androstene-3.beta.,17.beta.-diol, with a binding affinity that ranged from −6.1 kcal/mol to −6.5 kcal/mol. The inhibitory effect of *Saussurea costus* of SARS-CoV-2 entry into the cell was studied using a pseudovirus with Spike proteins from the D614G variant and the VOC variants Gamma and Delta. Based on the viral cycle of SARS-CoV-2, our results suggest that the *Saussurea costus* aqueous extract has no virucidal effect and inhibits the virus in the events after cell entry. Furthermore, the biological activity of the aqueous extract was investigated against HSV-1 virus and two bacterial strains, namely *Staphylococcus aureus* ATCC BAA 1026 and *Escherichia coli* ATCC 9637. According to this study, an enormous number of water-soluble inhibitors were identified from *Saussurea costus* against the M^pro^, and this is unprecedented as far as we know.

## 1. Introduction

Coronaviruses are a large family of single-stranded, enveloped, zoonotic RNA viruses, and some cause fewer mild disease, such as fevers and the common cold, than others [1,2,3,4]. However, other viruses cause more severe illnesses such as Middle East Respiratory Syndrome (MERS) and severe acute respiratory syndrome coronavirus 2 (SARS-CoV-2), and some of these viruses are easily transmitted from person to person, unlike other viruses [5,6]. It has been found that civet cats can transmit SARS-CoV to humans; MERS-CoV is transmitted between dromedary camels and humans, but SARS-CoV-2 was first found in seafood markets [7]. However, the origin of the SARS-CoV-2 virus is not known, and the two opposing theories that have recently dominated discussion are the “laboratory escape” hypothesis and zoonotic evolution [8].

The severe acute respiratory syndrome coronavirus was isolated from the airway epithelial cells of infected humans, and a phylogenetic analysis of full-length genome sequences obtained from infected patients revealed that SARS-CoV-2 is similar to SARS-CoV and uses the same cell entrance receptor, angiotensin-converting enzyme 2 [9]. Additionally, many mutants of the SARS-CoV-2 virus have been discovered, including the Omicron variant (B. 1.1.529) and the Delta variant (AY.3), which have high transmissibility rates [10,11,12]. As a consequence, they pose a huge threat to public health around the world [13,14,15]. The World Health Organization (WHO) has declared a medical emergency; scientific research is tackling this condition worldwide [16].

In the laboratory, drug-testing techniques assist scientists in identifying new and suitable candidates versus different convergent diseases [17]. However, developing drugs consumes time, money, humans, and specialized equipment. Therefore, many developing countries have resorted to using herbs to treat many diseases [18]. In ancient times and throughout history, plants were the main source of medicine and pharmaceuticals. However, modern medicine has mostly ignored medicinal plants [19]. Medicinal plants are widely used to treat many common diseases such as malaria, cholera, and asthma. Herbal medicine is considered to be the principal healthcare system of choice in various developing countries for several factors, including cost and the lack of availability of other systems. Antiviral plant compounds may work by inactivating virus particles, reducing endocytic activity, inhibiting viral enzymes and molecular reproduction mechanisms, altering virus capsid properties, blocking virus adsorption and penetration into human cells, inhibiting reverse transcriptase, inhibiting translation, reducing the expression level, and apoptosis [20]. Investigating the possible applications of medicinal plants could be beneficial for the control of the pandemic. Several studies have found that medicinal herbs such as Vernonia amygdalina, *Nigella sativa*, *Eurycoma longifolia*, and *Azadirachta indica* can assist in the prevention of and hasten the recovery from COVID-19 disease [21].

One of the essential herbs is *Saussurea costus*, which is widely used in Asia and the Arab world [22]. The plant is a member of the Asteraceae family, which is located worldwide; however, its most prevalent regions are India, Pakistan, and the Himalayas [23,24]. *Saussurea costus* is an herb used in many traditional medicine systems to treat asthma, inflammatory diseases, ulcers, and stomach problems [25,26]. Furthermore, several laboratory and animal model studies have demonstrated that *Saussurea costus* shows anti-inflammatory, anti-trypanosomal, potent anticancer, antibacterial, antifungal, and antiviral activity, confirming its tradition of use in various forms of medicine [26,27,28,29,30,31,32,33,34].

In spite of vaccination efforts and distinguished therapies for some viral illnesses, humans continue to lose the battle against viruses [35]. Therefore, for infectious diseases in particular, people need to go back to medicinal plants, which are a huge and all-natural drug store [36]. This study put a special emphasis on finding inhibitors against the main protease (M^pro^), as it is considered an ideal target for the treatment of COVID-19 [37,38,39,40,41,42]. The blind molecular docking approach was performed utilizing the elucidated compounds extracted in water at room temperature from *Saussurea costus*. It is indisputable that the inhibitor preference increases with increased solubility in water and answers why people in rural communities use this plant to prevent and treat COVID-19.

## 2. Materials and Methods

### 2.1. Cell Cultures and Plasmids

DMEM (Gibco, Grand Island, NY, USA) supplemented with 10% fetal bovine serum (Gibco), antibiotics (Sigma-Aldrich, Saint Louis, MO, USA), and non-essential amino acids (Gibco) was used to sustain HEK293T and stable HEK293T-ACE2-expressing cells at 37 °C and in a 5% CO_2_ environment. Puromycin was also present in the HEK293-ACE2 cells at a final concentration of 1 µg/mL. the following plasmids were used: pNL4.3-Env-FLuc, spike-G614-19, and pCMV-VSV-G [43].

### 2.2. Extraction and Preparation for GC-MS Analysis

*Saussurea costus* root was purchased from an herbal store in Riyadh, Saudi Arabia. An amount of 50 gm of dry *Saussurea* roots was extracted with 250 mL of distilled water for 96 h at room temperature using a multifunctional Orbital Shaker (BioSan PSU-20i). The supernatant was then filtered through filter paper, and the extract was allowed to dry. To 0.1 g of the extract, 1.5 g of anhydrous sodium sulfate was added and then dissolved with 10 mL of analytical-grade ethanol. The solution was then passed through a 0.45 mm syringe filter and into a 1.5 mL vial, where it was prepared for injection into GC-MS.

### 2.3. GC-MS Analysis Conditions

This study was carried out on samples through the use of the GM-MS technique model (GC-MS-QP2010-Ultra) with serial number 020525101565SA (Shimadzu, Kyoto, Japan), and a capillary column (Rtx-5 ms-30 m 0.25 mm 0.25 mm) manufactured by Restek, D-81379 Munich, German. The sample was injected using the split mode with helium as the carrier gas passing through at a flow rate of 1.61 mL/min. The temperature program was started at 50 °C with a rate of 10 °C per minute and ended at 300 °C with a hold time of 10 min. The injection port temperature was 300 °C, the ion source temperature was 200 °C, and the interface temperature was 250 °C. It took 35 min to analyze the sample using the scan mode in the *m*/*z* 40–500 charges to ratio range, with a run time of 40 min. The sample’s constituents were determined by evaluating their retention index and mass fragmentation patents to tose available in the National Institute of Standards and Technology library (NIST).

### 2.4. Molecular Docking

The crystal structure of the M^pro^ of SARS-CoV-2 (PDB ID: 6Y2E) was downloaded from the Protein Data Bank database. Then, water residues were removed before minimization for 1000 steepest descent steps at 20 conjugate gradient steps. The 3D structure of the screened compounds was generated as a PDB file utilizing SMILES strings and PubChem ID using the build structure function in UCSF Chimera which developed by Resource for Biocomputing, Visualization, and Informatics (RBVI) at the University of California, San Francisco, USA [44,45,46,47]. Their energy was minimized with an antechamber plugin in UCSF Chimera for 14,000 steepest descent steps at 8000 conjugate gradient steps. Molecular docking was accomplished using the AutoDock Vina bulging in UCSF Chimera [48]. A grid box of (–15 × −24 × 15) Å centered at (35, 65, 65) Å was used while maintaining the default parameter values. The predicted affinity score was explored through the UCSF Chimera View Dock tool. UCSF Chimera has been used to process and visualize images, hydrogen bonds, and van der Waals interactions [44,45,48,49,50,51].

### 2.5. Antibacterial Activity Evaluation

The disc diffusion assay was utilized to evaluate the extract’s initial antimicrobial activity against the selected microbial strains, including a Gram-positive bacterium (*Staphylococcus aureus* ATCC BAA 1026) and a Gram-negative bacterium (*Escherichia coli* ATCC 9637). The Kirby–Bauer disc diffusion susceptibility protocol was used with minor modifications [52]. Briefly, the aqueous crude extract was reconstituted in sterile distilled water to obtain a concentration of 100 mg/mL. Microbial strains were adjusted to 0.5 McFarland (10^8^ CFU/mL) suspension using normal saline (0.9% NaCl) and were directly swabbed onto nutrient agar plates (Oxoid, UK). An amount of 10 µL of the aqueous extract (100 mg/mL) was placed on sterile 6-millimeter paper discs and allowed to dry under aseptic conditions. A disc holding 10 µL of normal saline served as a negative control, while discs containing chloramphenicol (2.5 mg/mL) served as a reference drug (positive control), and the plates were incubated at ambient temperature for up to 18 h. Plates were then inspected for the inhibition zones around the discs and the test was repeated twice.

### 2.6. Cytotoxicity Assays

Cytotoxicity assays were performed as previously described [53]. Using the resazurin technique, the maximum non-toxic concentration was determined. In a 96-well plate, HEK293T-ACE2 cells were grown at a density of 1 × 10^4^ cells per well while being exposed to various doses of the natural products. Cells treated with the vehicle (DMSO) were utilized as a standard and control. At 48 h after resazurin addition, cell viability was assessed after 3 h. At 570 and 630 nm, the Multiskan TM GO (Thermo Scientific, Waltham, MA, USA) was used to detect absorbance. The MNTC has the highest concentration and less than 10% cytotoxicity.

### 2.7. Antiviral Activity against SARS-CoV-2

The assay was performed in HEK293-ACE2 cells as previously described [54]. Briefly, 1 × 10^4^ cells in suspension were added to each well of 96-well plates and infected in the presence of 2 mg/mL of the extract. Firefly luciferase activity was measured 48 h later using the Dual-Luciferase Reporter Assay System kit (Promega, Madison, WI, USA) and the Fluoroskan FL (Thermo Scientific). HEK293T-ACE2 cells transduced with the pseudotyped virus without the extract were used as untreated control cells. The following formula was used to calculate the percentage of inhibition: 100 − (RLUs of treated cells/RLUs of control untreated cells) × 100.

### 2.8. Time of Addition Assay of HSV-1

The time of addition assay was carried out in three different experimental conditions according to the time the extract was added. In the pre-infection condition, the extract was added two hours before viral adsorption. Subsequently, cells were washed with PBS, the viral inoculum was added, and the infection was performed as previously described. In the adsorption condition, the extract was added with the viral inoculum and was incubated for 1 h at 37 °C. After that, the viral inoculum was removed, cells were washed with PBS and replaced with DMEM 2%, and infection was performed as previously described. For the post-entry condition, the viral inoculum was added and incubated for 1 h at 37 °C, washed with PBS, and DMEM 2% with the extract was added. After 72 h, the virus genome in the supernatant was quantitated by qPCR, and the antiviral activity was quantified as previously described [54].

### 2.9. Statistical Analysis

The data of at least three separate experiments were provided as the mean and standard deviation (SD). Each group’s differences were evaluated through a *t*-test. Lines between the groups being compared represent statistically significant differences (* *p* = 0.05, ** *p* = 0.01, *** *p* = 0.001, and **** *p* = 0.001).

## 3. Results and Discussion

**Figure 1:**
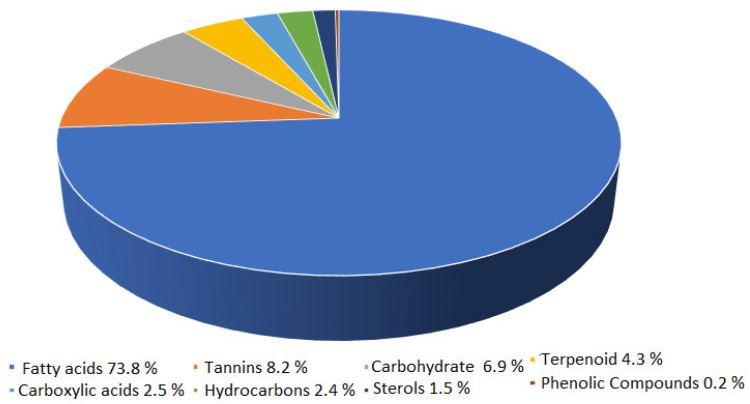
Phytochemicals in water-extracted compounds from *Saussurea costus* as identified by GC-MS.

Phytochemical analysis of an extract of the *Saussurea* root revealed the presence of forty-eight chemical substances, all of which exhibited specific mass spectral fragmentation characteristics similar to known compounds in the National Institute of Standards and Technology (NIST) library, as shown in Table S1. According to the data, fatty acids account for the highest percentage of phytochemicals at 73.8%, followed by tannins at 8.2%, carbohydrates at 6.9%, terpenoids at 4.3%, carboxylic acids at 2.5%, hydrocarbons at 2.4%, and sterols at 1.5%. Phenolic compounds account for the lowest percentage of phytochemicals, at 0.2%, as shown in Figure 1.

**Figure 2:**
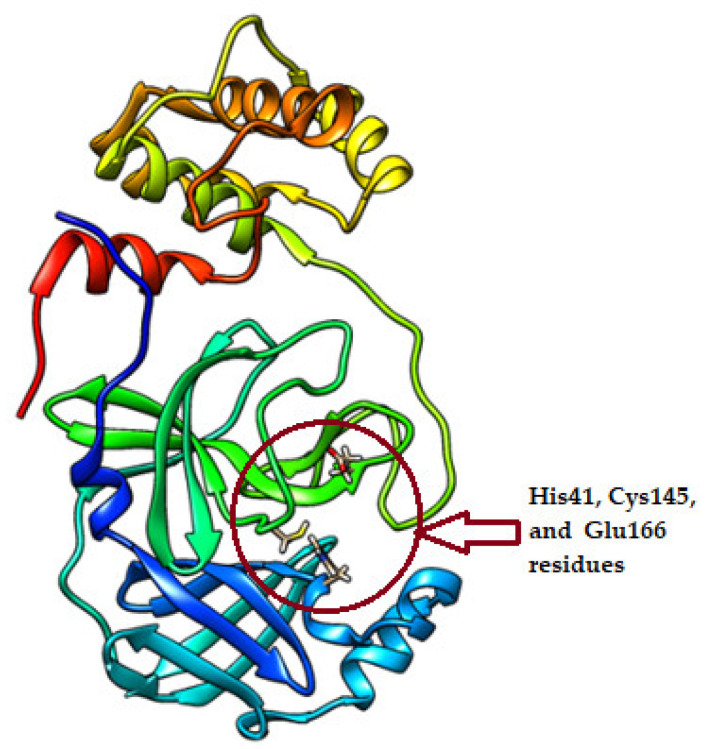
The crystal structure of the main protease of SARS-CoV-2 (PDB ID: 6Y2E).

**Figure 3:**
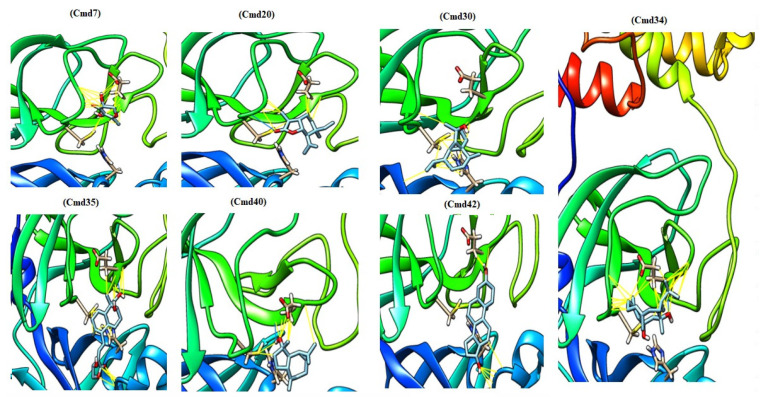
The active inhibiter docked with the active sites of the main protease of SARS-CoV-2.

The identified extracted compounds were used in blind docking experiments against the M^pro^, with a particular focus on the interaction with the catalytic dyad of His41 and Cys145 in addition to Glu166 residue (Figure 2). In Table 1, we present the results of the docking experiment, which clearly show the uniqueness of the water extract of *Saussurea costus*, as 22 of the 48 compounds showed the ability to interact with the active sites of the M^pro^. Of these compounds, eight were carbohydrates, five were fatty acids, three were terpenoids, two were carboxylic acids, one was a tannin, one was a phenolic compound, and one was a sterol. Figure 3 depicts the docked complexes of the top seven candidates. In Figure 3, the hydrogen bonds and van der Waals interactions that were formed between these screened compounds and the M^pro^ expected from the molecular docking were monitored. The compounds 4,8,13-Cyclotetradecatriene-1,3-diol-1,5,9-trimethyl-12-(1-methylethyl) (cmd34), Andrographolide (cmd35), and delta.4-Androstene-3.beta.,17.beta.-diol (cmd42) showed a higher binding affinity among the identified compounds, with the binding affinity ranging from −6.3 kcal/mol to −6.5 kcal/mol. They are a hydrocarbon, a terpenoid, and a sterol, respectively. These results indicate that *Saussurea costus,* which is used in traditional medicine as a COVID-19 remedy, produced an enormous number of inhibitors against the M^pro^ that were water soluble in green conditions. Therefore, the plant’s mixture of active components may have a more effective therapeutic impact than a single isolated chemical.

The literature has shown that tannins, terpenoids, and phenolic substances can inhibit SARS-CoV-2 [55,56,57,58]. In addition, it has been reported that the quinolin-2-carboxylic acids found in *Ephedra sinica* are being suggested as possible treatment agents for COVID-19 [59]. Fatty acids are self-defense agents in organisms and have a variety of biological actions, particularly anti-inflammatory properties [60]. Carbohydrates have not been demonstrated to have a therapeutic impact on their own, but they may boost the efficacy of the therapeutically essential components and be used to produce polysaccharide immunomodulatory with potential medicinal and vaccine applications [61,62]. Sterol molecules have exhibited a wide range of biological actions, the majority of which act as cancer inhibitors, anti-inflammatory agents, and immunomodulatory and anti-viral agents [63]. Some of the compounds isolated from this plant, including costsunolide, dehydrocostus lactone, and cynaropicrin, appear to be capable of being developed as bioactive molecules [64,65,66].

**Figure 4:**
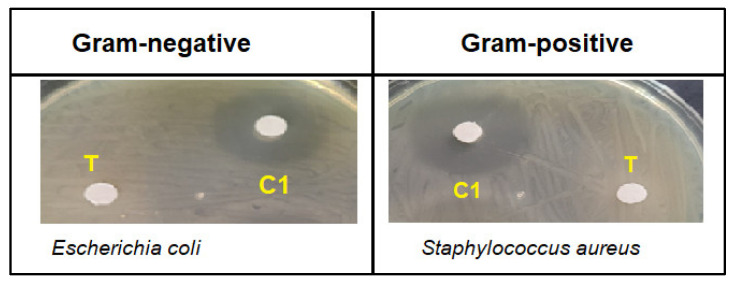
Negative zone of inhibition of *Saussurea costus* aqueous extract (100 mg/mL) (T) compared to chloramphenicol (2.5 mg/mL) (C1) against tested bacteria.

As shown in Figure 4, the results of the antibacterial evaluation of the crude aqueous extract of *Saussurea costus* showed no activity in an inhibition zone of less than 7.0 mm (the diameter of the paper disc is 6.0 mm). Previous studies reported that methanol and ethanol extracts have remarkable antibacterial activities against various Gram-positive and Gram-negative bacteria [67,68]. Our results are also in agreement with a previous study that revealed that the water root extract of *Saussurea costus* did not show antibacterial activity against a Gram-positive bacterium (*Bacillus subtilis*) and a Gram-negative bacterium (*Escherichia coli*); however, these results showed higher activity with the non-polar extract (Chloroform extract) [69], indicating that the bioactive phytochemical molecules were not attracted by the aqueous extract and that the bioactive compounds might have a non-polar or a hydrophobic nature.

**Figure 5:**
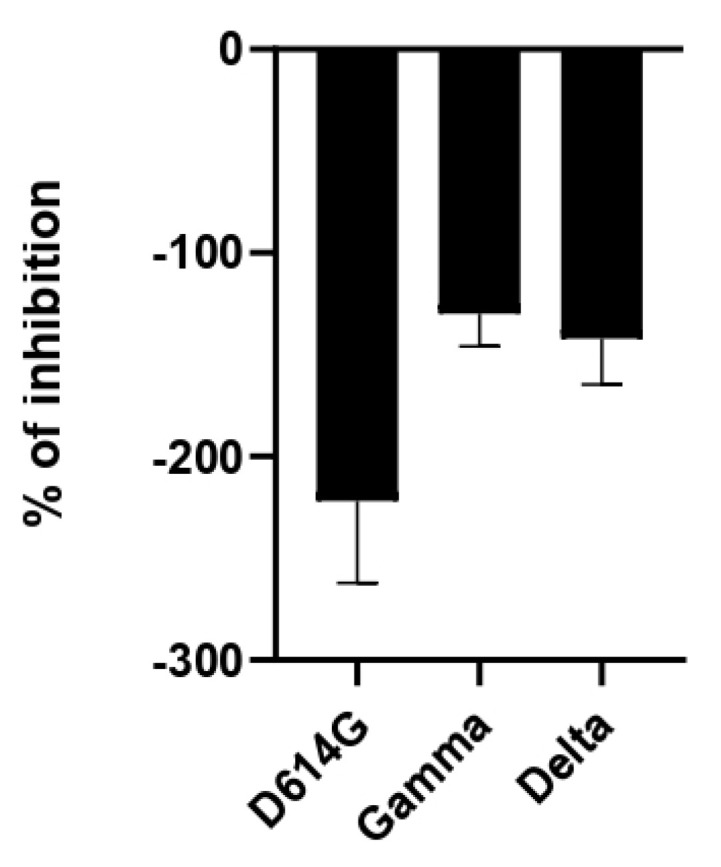
Antiviral activity of *Saussurea costus* aqueous extract against SARS-CoV-2. HEK−293T ACE2 cells were infected with the corresponding Spike-pseudotyped virus in the presence and absence of the extract. After 24 h, the luciferase activity was measured. The % of inhibition was determined as the ratio between treated and untreated cells.

To determine the antiviral activity against SARS-CoV-2, the inhibitory activity was evaluated using the pseudovirus method. This system allows for the study of inhibitors of virus entry into the cell [54]. The antiviral activity was performed using a pseudovirus with Spike proteins from the D614G variant and the VOC variants Gamma and Delta. As shown in Figure 5, no antiviral effect of the extract from *Saussurea costus* was observed, and unexpectedly, an increase in pseudovirus infection by the extract was observed. This effect was similar for all three SARS-CoV-2 variants. These results suggest that the extract from *Saussurea costus* facilitates the cell entry of SARS-CoV-2.

**Figure 6:**
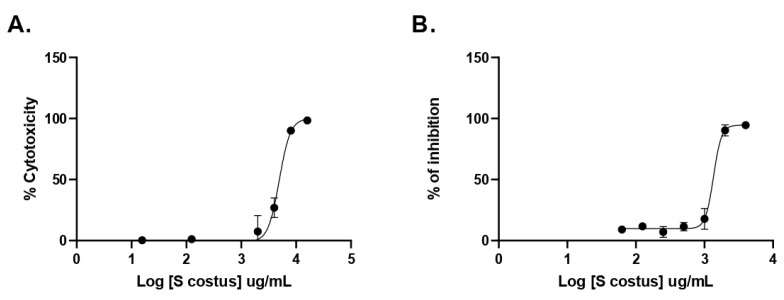
Antiherpetic activity and cytotoxicity of *Saussurea costus* aqueous extract. (**A**) Vero cells were incubated with increasing concentrations of the extract, and after 72 h, the cytotoxicity was measured as described in Section 2. (**B**) Vero cells were infected at MOI 1.5 with HSV-1 and incubated with increasing concentrations of the extract. After 72 h, the virus genome production was quantitated in the supernatant by qPCR. The % of inhibition was determined as the ratio between treated and untreated infected cells. Data are expressed as mean +/− SD for *n* = 2.

Also, the antiviral activity of the *S. costus* aqueous extract against HSV-1 was evaluated. For this purpose, serial dilutions of the extract were performed, and the amount of virus produced was evaluated by qPCR. As shown in Figure 6, the *Saussurea costus* aqueous extract showed antiviral activity with an effective concentration 50 (EC_50_) of 1.35 mg/mL. In addition, a cytotoxic concentration (CC_50_) of 4.92 mg/mL and a selectivity index of 3.6 were observed.

**Figure 7:**
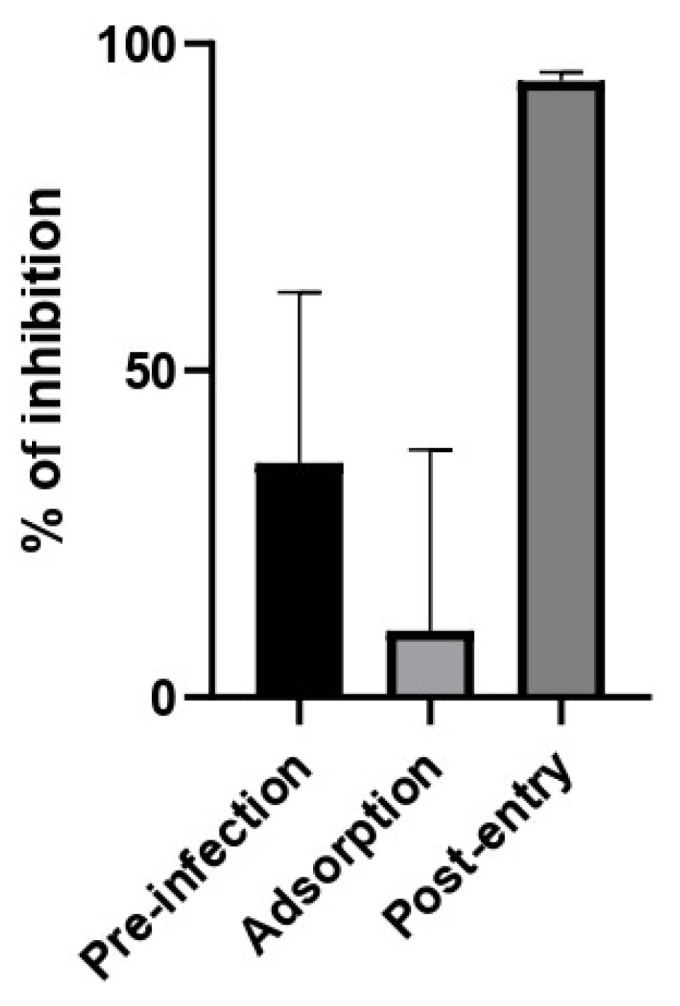
Time of addition assay of *Saussurea costus* aqueous extract. Vero cells were infected at MOI 1.5 and incubated with the extract at different times during the infection as is described in Section 2. After 24 h.p.i, the virus genome production was quantitated in the supernatant by qPCR. The % of inhibition was determined as the ratio between treated and untreated infected cells. Data are expressed as mean +/− SD for *n* = 3.

To obtain some information on the step of the viral cycle at which the extract exhibits antiviral activity, a time-of-infection study was performed. For this purpose, the extract was added at different times during infection. In the pre-infection condition, the extract was added 2 h prior to the addition of the virus and then removed to continue with the infection. In the adsorption condition, the virus was added together with the extract and incubated for one hour and then removed. In the post-entry condition, the extract was added after the virus entered the cell. As can be seen in Figure 7, the aqueous extract of *Saussurea costus* showed post-entry antiviral activity. This suggests that the *Saussurea costus* aqueous extract has no virucidal effect and inhibits the virus during the events after cell entry.

The information presented here describes the chemical profiles of *Saussurea costus* derived from a green extraction procedure using a water-based solvent at ambient temperature. Eighty-four compounds were identified, including fatty acids, tannins, carbohydrates, terpenoids, carboxylic acids, hydrocarbons, phenolic compounds, and sterols. The inhibitory effect of *Saussurea costus* on SARS-CoV-2 entry into the cell was studied using a pseudovirus with Spike proteins from the D614G variant and the VOC variants Gamma and Delta. Furthermore, the inhibition of the M^pro^ by these compounds was investigated using the molecular docking approach. Twenty-two candidates showed a good affinity to bind with the active site of the M^pro^. According to this study, twenty-two candidates were identified from *Saussurea costus* against the M^pro^, which has been used in traditional medicine as a COVID-19 remedy. The number of water-soluble inhibitors that were identified from *Saussurea costus* against the M^pro^ is unprecedented to our knowledge.

## Figures and Tables

**Table 1:** The binding affinity of the extracted compounds active against 3-chymotrypsin-like protease (3CL^pro^).

Compound ID/Class	Structure	Binding Affinity (Kcal/Mol)/(RMSD)	Hydrogen Bond	van der Waals
Cmd1/carbohydrate	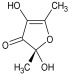	−4.7/ (0.00–3.2)	GLU 166, HIS 164, HIS 163	HIS 164, MET 165, HIS 163, LEU 141, ASN 142, GLU 166, PHE 140, SER 144, CYS 145
Cmd2/carbohydrate	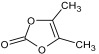	−3.9/ (40.82–41.72)	HIS 163	HIS 163, MET 165, GLU 166, SER 144, ASN 142, HIS 164, PHE 140
Cmd3/carbohydrate	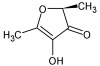	−4.3/ (40.10–41.20)	HIS 163	GLU 166, HIS 163, MET 165, HIS 164, ASN 142, LEU 141, PHE 140, CYS 145
Cmd5/carbohydrate	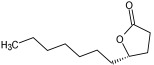	−4.4/ (27.24–29.57)	HIS 163, GLU 166	HIS 163, MET 49, GLU 166, MET 165, HIS 41, GLN 189
Cmd6/carboxylic acid	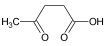	−3.8/ (25.23–26.15)	HIS 163, GLU 166, PHE 140	GLU 166, MET 165, CYS 145, HIS 163, ASN 142, SER 144, PHE 140, LEU 141
Cmd7/carbohydrate	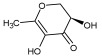	−5.0/ (0.00–0.00)	HIS 163	HIS 163, GLU 166, PHE 140, MET 165, HIS 164, CYS 145, ASN 142, SER 144, LEU 141
Cmd8/fatty acid	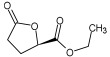	−4.3/ (40.97–42.42)	HIS 163, GLU 166	HIS 163, GLU 166, MET 165, HIS 164, PHE 140, MET 49, CYS 145, ASN 142, SER 144, LEU 141
Cmd11/carbohydrate	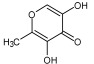	−4.9/ (44.57–45.67)	-	HIS 163, GLU 166, MET 165, HIS 164, PHE 140, LEU 141, CYS 145, ASN 142, SER 144
Cmd12/tannins	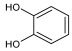	−4.6/ (44.33–45.19)	HIS 164	HIS 163, HIS 164, MET 165, GLU 166, PHE 140, LEU 141, ASN 142, SER 144, CYS 145
Cmd13/carbohydrate	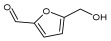	−4.3/ (24.50–25.23)	PHE 140, HIS 163	HIS 163, HIS 164, MET 165, GLU 166, PHE 140, LEU 141, ASN 142, SER 144, CYS 145, HIS 41
Cmd15/fatty acid	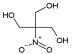	−4.2/ (25.99–27.47)	HIS 163	HIS 163, HIS 164, MET 165, GLU 166, PHE 140, LEU 141, GLY 143, ASN 142, SER 144, CYS 145
Cmd18/Phenolic Compound	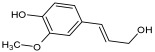	−4.9/ (42.17–45.62)	HIS 164, GLU 166	GLN 189, PRO 168, THR 190, HIS 164, MET 165, GLU 166, MET 49, HIS 41, CYS 145
Cmd20/carbohydrate	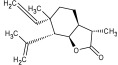	−5.1/ (29.29–33.07)	GLY 143	MET 49, GLU 166, ASN 142, GLY 143, GLN 189, MET 165, LEU 141, HIS 41, CYS 145
Cmd21/Carboxylic acid	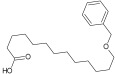	−4.3/ (28.11–30.02)	GLU 166, GLY 143	GLY 143, ASN 142, MET 49, THR 26, LEU 27, HIS 41, HIS 164, GLU 166, GLN 189, MET 165, CYS 145
Cmd26/Fatty acid	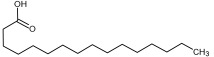	−4.2/ (27.28–29.77)	HIS 163, SER 144	SER 144, HIS 163, THR 25, LEU 27, GLU 166, GLN 189, ASN 142, MET 49, PHE 140, LEU 141
Cmd30/Terpenoid	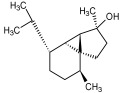	−5.2/ (29.65–31.24)	-	MET 49, THR 25, THR 26, LEU 27, HIS 41, ASN 142, CYS 145, HIS 164
Cmd34/Hydrocarbon	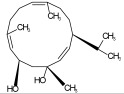	−6.3/ (42.35–46.32)	GLU 166	GLU 166, ASN 142, PRO 168, PHE 140, LEU 141, HIS 163, MET 165, ASN 142, CYS 145, LEU 167, HIS 164, GLN 189
Cmd35/Terpenoid	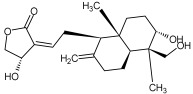	−6.3/ (30.17–32.16)	GLU 166	THR 25, MET 165, GLU 166, GLN 189, ASN 142, THR 45, SER 46, MET 49, CYS 44, HIS 41, CYS 145, THR 24
Cmd38/Fatty acid	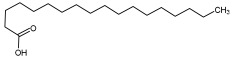	−3.7/ (26.63–30.65)	HIS 163	HIS 163, GLU 166, MET 49, SER 144, GLN 189, PHE 140, LEU 141, ASN 142, HIS 41, HIS 164, MET 165
Cmd40/Terpenoid	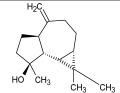	−5.4/ (43.11–45.74)	HIS 164	GLU 166, MET 49, GLN 189, ASN 142, MET 165, CYS 145, HIS 164
Cmd41/Fatty acid	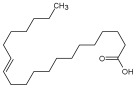	−4.3/ (28.45–31.03)	HIS 163, PHE 140	HIS 163, ASN 142, SER 144, LEU 141, PHE 140, LEU 27, GLN 189, MET 49, THR 25, THR 26, GLU 166, CYS 145, GLY 143, HIS 41, MET 165
Cmd42/Sterol	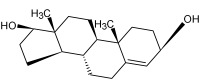	−6.5/ (30.43–33.11)	-	MET 49, HIS 163, ASN 142, SER 144, LEU 141, PHE 140, LEU 27, GLN 189, THR 25, THR 26, GLU 166, CYS 145, GLY 143, HIS 41, MET 165

## Data Availability

The data will be available upon suitable request.

## Supplementary Materials

The following supporting information can be downloaded at: https://www.mdpi.com/article/10.3390/molecules27154908/s1, Table S1: GC-MS discrimination of phytochemicals from the water extract of *Saussurea costus*.

## Sample Availability

Samples of the compounds extracted from *Saussurea costus* are available from the authors.
