# Supplementary material for: Phytochemical Discrimination, Biological Activity and Molecular Docking of Water-Soluble Inhibitors from Saussurea costus Herb against Main Protease of SARS-CoV-2
Source: Molecules. 2022 Aug 1;27(15):4908. doi: 10.3390/molecules27154908 (PMC9370511; doi:10.3390/molecules27154908)
Supplement: Supplementary file 1 [file molecules-27-04908-s001.zip › molecules-1666732-supplementary.pdf]

Phytochemical discrimination and molecular docking of water-soluble inhibitors from *saussurea costus* herb against main protease of SARS-CoV-2

Hajo Idriss [1,2], Babeker Siddig [3,4], Pamela Gonzalez Maldonado [5], H. M. Elkhair [1], A. I. Alakhras [1,6], Emad M. Abdallah [7], Pablo Hernán Sotelo[5] and Amin O. Elzupir [1,2,*]

[1] Deanship of Scientific Research, Imam Mohammad Ibn Saud Islamic University (IMSIU), P.O. Box 90905 Riyadh, 11623 Saudi Arabia; hiidriss@imamu.edu.sa (H.I.); hmdirar@imamu.edu.sa (H.M.E.); aakhrasi@imamu.edu.sa (A.I.A.)
[2] Department of Physics, College of Science, Imam Mohammad Ibn Saud Islamic University (IMSIU), P.O. Box 90905 Riyadh, 11623 Saudi Arabia
[3] Alawia Imam Institute for Pharmaceutical Research and Development, University of Medical Science and Technology, Khartoum,11115 Sudan; gcfid@savola.com
[4] Savola Edible Oils , Khartoum,11115 Sudan
[5] Biotechnology Department, Facultad de Ciencias Químicas, Universidad Nacional de Asunción, San Lorenzo 111421, Paraguay; pame.54.pg12@gmail.com (P.G.M.); phsotelo@qui.una.py (P.H.S)
[6] Department of Chemistry, College of Science, Imam Mohammad Ibn Saud Islamic University (IMSIU), Riyadh, Saudi Arabia
[7] Department of Science Laboratories, College of Science and Arts, Qassim University, Ar Rass 51921, Saudi Arabia; emad100sdl@yahoo.com
* Correspondence: aoalamalhuda@imamu.edu.sa

Table S1. GC-MS discrimination of phytochemicals from water-extract of *saussurea costus*.

No	Compound name	Ret.Time	Area	Area%	Classification
1.	2,4-Dihydroxy-2,5-dimethyl-3(2H)-furan-3-one	3.565	835809	0.73	Carbohydrate
2.	1,3-Dioxol-2-one,4,5-dimethyl-	4.256	1107090	0.96	Carbohydrate
3.	2,5-Dimethyl-4-hydroxy-3(2H)-furanone	4.516	2073246	1.80	Carbohydrate
4.	Maltol	4.758	2641563	2.30	Tannins
5.	2(3H)-Furanone, 5-heptyldihydro-	4.916	620539	0.54	Carbohydrate
6.	Pentanoic acid, 4-oxo-	5.180	1205524	1.05	Carboxylic acid
7.	4H-Pyran-4-one, 2,3-dihydro-3,5-dihydroxy-6-methyl-	5.605	8504711	7.40	Carbohydrate
8.	5-Oxotetrahydrofuran-2-carboxylic acid, ethyl ester	5.843	277841	0.24	Fatty acid
9.	Benzoic acid	5.943	218627	0.19	Carboxylic acid
10.	2-Hexanone, 3,3-dimethyl-	6.000	286844	0.25	Hydrocarbon

11.	4H-Pyran-4-one, 3,5-dihydroxy-2-methyl-	6.168	329914	0.29	Carbohydrate
12.	Catechol	6.440	489444	0.43	Tannins
13.	5-Hydroxymethylfurfural	6.502	3022022	2.63	Carbohydrate
14.	1,2,3-Propanetriol, 1-acetate	6.736	3959968	3.45	Tannins
15.	Cyclobutanecarboxylic acid, 2-tridecyl ester	7.417	2788842	2.43	Fatty acid
16.	1,3-Propanediol, 2-(hydroxymethyl)-2-nitro-	9.583	10089211	8.78	Tannins
17.	4,5-Heptadien-2-one, 3,3,6-trimethyl-	12.618	886867	0.77	Terpenoid
18.	4-((1E)-3-Hydroxy-1-propenyl)-2-methoxyphenol	12.826	131120	0.11	Phenolic Compound
19.	Tetradecanoic acid	13.335	291448	0.25	Fatty acid
20.	2(3H)-Benzofuranone, 6-ethenylhexahydro-3,6-dimethyl-7-(1-methylethenyl)-, [3S-(3.alpha.,3a.alpha.,6.alpha.,7.beta.,7a.beta.)]-	13.605	170727	0.15	Carbohydrate
21.	Benzyl oxy tridecanoic acid	14.080	88542	0.08	Carboxylic acid
22.	Tricyclo[4.2.2.0(2,5)]dec-7-ene, 7-(5-hexynyl)-	14.288	143374	0.12	Terpenoid
23.	.alpha.-Bulnesene	14.411	532204	0.46	Terpenoid
24.	Cyclodecacyclotetradecene, 14,15-didehydro-1,4,5,8,9,10,11,12,13,16,17,18,19,20-tetradecahydro-	14.982	1504459	1.31	Hydrocarbon
25.	(-)-Spathulenol	15.068	906977	0.79	Terpenoid
26.	n-Hexadecanoic acid	15.386	6325410	5.50	Fatty acid
27.	Bicylo[4.1.0]heptane, 7-bicyclo[4.1.0]hept-7-ylidene-	15.483	672778	0.59	Terpenoid
28.	Pregnan-20-one, 3-hydroxy-	15.828	429400	0.37	Sterol
29.	Caryophyllene oxide	15.946	218763	0.19	Terpenoid
30.	4-epi-cubedol	16.118	323009	0.28	Terpenoid
31.	1,2-15,16-Diepoxyhexadecane	16.475	177023	0.15	Hydrocarbon

32.	9,12-Octadecadienoic acid (Z,Z)-, methyl ester	16.605	628109	0.55	Fatty acid
33.	9-Octadecenoic acid (Z)-, methyl ester	16.685	556434	0.48	Fatty acid
34.	4,8,13-Cyclotetradecatriene-1,3-diol, 1,5,9-trimethyl-12-(1-methylethyl)-	16.769	374016	0.33	Hydrocarbon
35.	Andrographolide	16.844	105269	0.09	Terpenoid
36.	9,12-Octadecadienoic acid (Z,Z)-, methyl ester	16.980	25545692	22.23	Fatty acid
37.	9-Octadecenoic acid, (E)-	17.055	26043197	22.67	Fatty acid
38.	Octadecanoic acid	17.257	2943518	2.56	Fatty acid
39.	Linoleic acid ethyl ester	17.695	3445088	3.00	Fatty acid
40.	(-)-Spathulenol	18.039	466724	0.41	Terpenoid
41.	cis-13-Eicosenoic acid	18.733	133887	0.12	Fatty acid
42.	.delta.4-Androstene-3.beta.,17.beta.-diol	19.351	185696	0.16	Sterol
43.	(Z)6,(Z)9-Pentadecadien-1-ol	19.703	393634	0.34	Terpenoid
44.	9-Octadecenoic acid, 1,2,3-propanetriyl ester, (E,E,E)-	19.759	270387	0.24	Fatty acid
45.	Hexadecanoic acid, 2-hydroxy-1-(hydroxymethyl)ethyl ester	19.967	653760	0.57	Fatty acid
46.	Docosanoic acid	20.546	918087	0.80	Fatty acid
47.	Phenol, 2,6-dimethoxy-4-(2-propenyl)-	21.201	119649	0.10	Phenolic Compound
48.	.beta.-Sitosterol	26.024	876719	0.76	Sterol
